# Addiction Consultation Service: Effects on Return Visits to the Emergency Department

**DOI:** 10.7759/cureus.87070

**Published:** 2025-06-30

**Authors:** Grace VanGorder, Courtney Fraser, Lena Becker, Donald Dissinger, Sarah Kawasaki, Catherine A Marco

**Affiliations:** 1 Emergency Medicine, Penn State College of Medicine, Hershey, USA; 2 Psychiatry, Penn State College of Medicine, Hershey, USA; 3 Emergency Medicine, Penn State Health Milton S. Hershey Medical Center, Hershey, USA

**Keywords:** addiction, alcohol, opioids, psychiatry, substance use, treatment

## Abstract

Introduction

The Addiction Consultation Service of Hershey Medical Center offers assessment, treatment and outpatient resources to patients diagnosed with a substance use disorder. This study aims to evaluate the efficacy of this program in connecting patients to resources and to compare rates of return to the Emergency Department (ED) within 90 days of discharge between those consenting versus declining an addiction consult.

Methods

The Addiction Consult Service’s database was used to identify all patients who were evaluated between February 2023 and September 2023 for substance-related concerns in the ED.

Results

For the study, 258 eligible subjects were identified. The majority of patients (n=167/258, 65.3%) were admitted to inpatient hospital services, 21.8% (n=56/258) were discharged and 8.2% (n=21/258) were transferred to an inpatient drug and alcohol rehabilitation facility. Of them, 202/227 (89.0%) patients received a consultation from the Addiction Consult Service, and 25/227 (11.0%) received a consultation from another service. Of these patients, the majority consented to the treatment plan suggested by the Addiction Consult Service (n=148/202, 73.3%). Patients who consented to the treatment plan were less likely to return to the ED for substance use concerns within 30 days of discharge, compared to those who declined a consultation (*X*^2^(2, N=202)=24.1, *p*<0.0001). This finding was not significant within 30-90 days of discharge (*X*^2^(2, N=202)=4.5, *p*=0.1043).

Conclusions

Receiving an in-hospital consultation from an addiction specialty service was associated with fewer repeat ED visits related to substance use disorder within 30 days of discharge. Targeted follow-up and comprehensive treatment plans are likely needed to reduce repeat ED visits.

## Introduction

Substance use disorders (SUDs) pose a significant burden on the U.S. healthcare system, as well as society at large. According to the National Institute on Alcohol Abuse and Alcoholism, emergency department (ED) visits with a primary concern related to drug or alcohol use account for 4.58 percent of all ED visits in the United States annually [1]. This translates to approximately 6.3 million drug- and alcohol-related ED visits each year [2]. Beyond the strain on emergency services, the repercussions of SUDs extend into other areas of public health and social well-being. Individuals with SUD are at a significantly increased risk for co-occurring mental health conditions, including depression, anxiety, and post-traumatic stress disorder. These comorbidities often complicate treatment and recovery efforts and can lead to increased rates of hospitalization and disability.

SUDs are also strongly associated with elevated suicide risk. Studies have shown that people with alcohol use disorder are more likely to die by suicide compared to the general population, with similar patterns observed for opioid and stimulant use disorders [3]. The impact of SUDs on employment is equally concerning, as substance use is linked to increased absenteeism, decreased productivity, workplace injuries, and job loss. This not only affects the individual but also can contribute to economic losses for employers and the national economy.

Moreover, substance use can severely disrupt interpersonal relationships and family dynamics, leading to marital conflict, child neglect or abuse, and social isolation. Children of parents with SUDs are particularly vulnerable, facing a higher risk of developing emotional, behavioral, and academic problems [4]. The cumulative effects of SUDs further emphasize the urgent need for comprehensive prevention, treatment, and support systems across multiple sectors of society.

There are two major evidence-based models in which ED providers can provide treatment resources to patients treated for SUD [5]. The Bridge model is an evidence-based model in which emergency medicine doctors evaluate patients for opioid use disorder, refer the patient to an outpatient provider associated with their hospital system and provide a short-term buprenorphine prescription. The ED-Bridge model builds upon the Bridge model while incorporating dual-trained emergency medicine and addiction medicine physicians to provide greater continuity of care. These physicians offer longitudinal care by evaluating patients during the ED visit and maintaining clinical follow-up throughout the early treatment process.

In 2019, the Department of Psychiatry at Penn State Health Milton S. Hershey Medical Center launched a hospital-based Addiction Consult Service (ACS) that emulates several principles of the ED-Bridge model. However, the model employed at Penn State differs in several important respects. Unlike the formal ED-Bridge model, the consultation service is staffed by providers from the Department of Psychiatry and not by clinicians dual-trained in emergency and addiction medicine. As such, while the program supports ED-based initiation of treatment for SUDs, it does not meet the strict definition of an ED-Bridge model as originally conceptualized.

The ACS is composed of a multidisciplinary team that includes psychiatrists, internal medicine physicians, addiction medicine fellowship-trained physicians, advanced practice providers (APPs), psychiatry residents, social workers and certified recovery specialists. The team provides in-person consultations for patients presenting with substance use-related concerns Monday through Friday, 8 a.m. to 5 p.m. Consults involve assessment for SUDs, initiation of short-term medications for opioid use disorder (MOUD) pharmacotherapy with buprenorphine, naltrexone or methadone, and linkage to outpatient services. Outside of operating hours, peer recovery specialists are dispatched by the Dauphin County Department of Drug and Alcohol to assist with transfers to inpatient rehabilitation centers or outpatient services. Just for Today Recovery and Veteran Support Service of the Dauphin County Department of Drug and Alcohol supplements this work through their Warm Handoff program. This initiative aims to improve the health outcomes for patients who receive emergency medical care for opioid overdose by facilitating a seamless transition from the ED setting to specialty SUD treatment. This program differs in that it is not staffed by clinicians or employees of the hospital system. 

One system to monitor the efficacy of SUD treatment is through direct contact with the patients after their treatment course has been completed. This method has been found to be impractical, as it is difficult to make contact with patients after they have been discharged [6]. An indirect method of monitoring treatment efficacy is by reviewing hospital records to evaluate if and when patients return to the ED for a subsequent intoxication. The Hennepin County Medical Center in Minneapolis utilized this approach using a retrospective chart review to identify “frequent flyers” in their ED. They identified 325 of these patients out of 11,370 visits associated with alcohol intoxication over four years [7]. They found that these patients were more likely to have comorbid psychological disorders than patients who did not return for additional treatment of drug or alcohol intoxication. This study did not evaluate the impact of receiving SUD treatment between ED visits on future substance use. 

This study was undertaken to evaluate the efficacy of initiating treatment for SUD prior to discharge from Hershey Medical Center Emergency Department or inpatient units, and to determine the rate of subsequent ED visits following treatment. 

## Materials and methods

This study was submitted to the Penn State University Institutional Review Board (IRB) and determined to be exempt from the formal IRB review process. The Penn State Health Addiction Consult Service’s database was used to identify all patients who were evaluated between February 2023 and September 2023 for substance-related concerns. This database was created by the ACS to log all the consult requests they received since initiation of the program. Substance-related concerns were defined as presentations involving acute intoxication, withdrawal, or other trauma or clinical symptoms directly attributable to recent substance use, as determined by the evaluating clinician.

Patients were included if they were 18 years of age or older and presented to the Hershey Medical Center Emergency Department with a primary concern of substance intoxication or withdrawal. Inclusion was not dependent on the presence of a urine drug screen, though documentation of recent use and/or clinical assessment indicating intoxication or withdrawal was required. Hospital encounters within the selected timeframe were reviewed, and data were extracted via chart review by trained research assistants. ED utilization was quantified as the number of unique encounters within 0 to 90 days following discharge from the index encounter. We identified short-term returns within 30 days of discharge and delayed returns 30-90 days after discharge. These subsequent encounters were briefly reviewed to determine whether they were related to substance use. Encounters unrelated to substance use were excluded from this analysis.

Statistical analysis

Descriptive statistics were used to summarize participant characteristics. Categorical variables were reported as counts and percentages, and continuous variables were presented as means±standard deviations. Comparisons between groups were conducted using the chi-square test for categorical variables. A p-value <.05 was considered statistically significant. All statistical analyses were performed using SAS software, version 9.4 (SAS Institute, Cary, NC, USA).

## Results

Characteristics of study patients

Among the 258 eligible subjects, the majority were men (n=163/258, 63.2%), White (n=200/258, 77.5%), and not Hispanic/Latino (n=229/253, 90.5%) (Table 1). Most patients arrived as walk-ins (n=153/243, 63.0%).

**Table 1:** Demographic Information of 258 Patients Note: Data are presented as n (%) for categorical variables and mean ± standard deviation (SD) for continuous variables.

	n (%)
Gender	
Male	163 (63.2)
Female	95 (36.8)
Race	
Asian	8 (3.1)
Black	18 (7.0)
Other	32 (12.4)
White	200 (77.5)
Ethnicity	
Hispanic/Latino	24 (9.5)
Not Hispanic/Latino	229 (90.5)
Age M (SD)	38.0 (14.9)

Patterns of substance use among study participants

The most used substance reported was alcohol, followed by opioids (Table 2). Most patients reported last use of a substance was the day of arrival to the ED (n=176/255, 69.0%) or within the past week (n=77/255, 30.2%), and frequency of use was most often daily (n=215/239, 90.0%). Nearly half of the patients had a history of polysubstance use (n=123/253, 48.6%).

**Table 2:** Substance of Intoxication or Withdrawal Among Study Patients Note: Data are presented as n (%) for categorical variables.

	n (%)
Alcohol	175 (67.8)
Amphetamines	25 (9.7)
Cocaine	22 (8.5)
Opioids	63 (24.2)
Cannabis	15 (5.8)
Other substances	35 (13.6)

Services provided after ED arrival

Some patients (n=26/258, 10.1%) were activated as a trauma alert. Of the patients who came to ED, 167/256 patients (65.2%) were admitted to the hospital, 56/256 (21.8%) were discharged home, and 21/256 (8.2%) were directly transferred to an inpatient substance use treatment unit. The majority of patients received consults from either the addiction medicine or Just For Today consultation teams, with services recommended outlined in Table 3. Only 32% (n=75/234) of the patients were treated medically by the Addiction Consult Service for intoxication or withdrawal during their hospital stay. The majority were provided with recommendations for treatment plans after discharge.

**Table 3:** Addiction Medicine or Just for Today Consults and Services Recommendations Note: Data are presented as n (%) for categorical variables.

	n (%)
Consultation type	
Addiction medicine	202 (78.3)
Just for Today	25 (9.7)
No consult	31 (12.0)
Services recommended	
Inpatient	84 (41.6)
Inpatient and outpatient	12 (5.9)
Outpatient	106 (52.5)
Type of service recommended	
Medication	41 (22.5)
Medication and referral	75 (41.2)
Referral	66 (36.3)

Rate of return to the emergency department

Patients who received a consultation were less likely to return to the ED within 30 days of discharge, compared to those who declined a consultation. This finding was not significant in the 30-90 days post-discharge period. The type of consultation received (addiction medicine vs Just For Today vs none) did not impact return rates within 30 or 30-90 days from discharge (Table 4). There was also no statistically significant difference in the rate of return for patients who initiated medication management versus referral to resources alone (Table 5). Demographic variables of age, gender, race, and ethnicity did not result in a statistically significant different rate of return to the ED within 30 or 30-90 days (Table 6). Mode of arrival and insurance were not associated with the rate of return to the ED.

**Table 4:** Associations between Emergency Department Return Visits and Consults Note: Comparison of categorical variables between groups. Statistical analysis was performed using the chi-square test. Statistical significance was defined as p<0.05. n.s. = non-significant.

	n (%)	χ^2^	p
Received a consult	227	-	-
Return visit within 30 days	24 (10.6)	24.15	< .0001
Return visit at 30-90 days	41 (18.1)	4.52	n.s.
Type of consultation received			
Return visit within 30 days	55	1.12	n.s.
Just for Today	7 (28.0)		
Addiction medicine consult	48 (23.3)		
Return visit at 30-90 days	70	2.15	n.s.
Just for Today	9 (36.0)		
Addiction medicine consult	61 (30.0)		

**Table 5:** Associations Between Emergency Department (ED) Return Visits within 30 days and Type of Service Recommended Note: Data are presented as n (%) for categorical variables.

	Number of Repeated ED Visits in 30 Days, n (%)						
	0	1	2	3	4	5	Total
Type of service							
Medication	32 (78.05)	7 (17.07)	2 (4.88)	0 (0.00)	0 (0.00)	0 (0.00)	41
Medication and referral	58 (77.33)	14 (18.67)	2 (2.67)	0 (0.00)	1 (1.33)	0 (0.00)	75
Referral	49 (74.24)	13 (19.70)	3 (4.55)	0 (0.00)	0 (0.00)	1 (1.52)	66
Total	139	34	7	0	1	1	182

**Table 6:** Associations between ED Return Visits and Patient Variables Notes: Comparison of categorical variables between groups. Statistical analysis was performed using the chi-square test. Statistical significance was defined as p<0.05. n.s. = non-significant.

			Return visit within 30 days			Return visit at 30-90 days	
		n (%)	χ^2^	p	n (%)	χ^2^	p
Demographics							
Gender			2.94	n.s.		1.62	n.s.
	Male	36 (22.1)			46 (28.2)		
	Female	26 (27.4)			33 (24.7)		
Race			0.96	n.s.		0.21	n.s.
	Caucasian	37 (18.5)			60 (30.0)		
	Non-Caucasian	15 (25.9)			19 (32.8)		
Ethnicity			4.29	n.s.		2.84	n.s.
	Hispanic or Latino	7 (29.2)			10 (41.7)		
	Not Hispanic or Latino	54 (24.5)			68 (29.7)		
Mode of Arrival			2.59	n.s.		1.83	n.s.
	Self		40 (26.1)			50 (32.7)	
	Ambulance		17 (18.9)			23 (25.6)	
Insurance Status			6.52	n.s.		5.79	n.s.
	Private	23 (24.5)			20 (31.9)		
	Medicaid	20 (23.3)			22 (25.6)		
	Medicare	13 (34.2)			15 (39.5)		
	No Insurance	4 (12.5)			12 (27.5)		

## Discussion

Emergency department visits

Given the high annual burden of drug and alcohol-related emergency visits, it is imperative for healthcare systems to better understand opportunities for improved delivery of SUD care. This study allows us to analyze the strength of consultation systems in improving patient health outcomes, notably the service provided at Hershey Medical Center.

Our findings suggest that patients who consented to a treatment plan with the Addiction Consult Service or Just for Today consultation service were less likely to have a repeat ED visit within 30 days compared to patients who declined a consultation. The majority of patients consulted were recommended to pursue outpatient services (n=106/202, 52.4%), followed by inpatient services (n=84/202, 41.6%), and then a combination of the two services (n=12/202, 5.9%) (Table 7). Additionally, in terms of services received, the majority of patients were given medication and a referral (n=75/182, 41.2%), followed by medication alone (n=41/182, 22.5%) and referral alone (n=66/182, 36.3%) (Table 5). This data highlights the strength in providing outpatient service opportunities for patients struggling with substance use who are presenting to the ED. Our findings also suggest a benefit regarding medication initiation for substance use during times of acute crisis. Identifying what resources provided these patients with the greatest benefit allows for improvements in the standardization of patient care as well as resource allocation.

**Table 7:** Associations between Emergency Department (ED) Return Visits within 30 Days and Services Recommended Note: Data are presented as n (%) for categorical variables.

	Number of Repeated ED Visits in 30 Days, n (%)						
	0	1	2	3	4	5	Total
Services recommended							
Inpatient	59 (70.24)	20 (23.81)	3 (3.57)	0 (0.00)	1 (1.19)	1 (1.19)	84
Inpatient and outpatient	8 (66.67)	3 (25.00)	1 (8.33)	0 (0.00)	0 (0.00)	0 (0.00)	12
Outpatient	87 (82.08)	12 (11.32)	7 (6.60)	0 (0.00)	0 (0.00)	0 (0.00)	106
Total	154	35	11	0	1	1	202

Furthermore, our data strongly suggests the benefit that Addiction Medicine Consult services have on ED visits. Various studies have demonstrated the positive impact that case coordination within and between healthcare teams has in reducing substance use-related ED visits [8]. A multidisciplinary approach to patient care offers a benefit to patients struggling with substance use.

Within the realm of addiction medicine, personalized practice behaviors can play a crucial role in preventing return to use [8]. Furthermore, treating substance use through valuable paradigms like the ED-Bridge model and similar variations has the potential to reduce healthcare costs. Studies have shown that addressing SUD with integrated treatment modalities can play a role in both individual and societal savings [9]. Consultation requests were recorded by the Addiction Consultation Service starting in late 2019. The review of data in the past four years reveals that there were 538 consultation requests in 2020, 648 in 2021, 580 in 2022, 579 in 2023 and so far in 2024 (as of September 12, 2024) numbered 347 revealing what appears to be a down trend in overall consultation requests although with an apparent increase in complexity and presence of polysubstance use and treatment, according to Addiction Consult Service member reports. Since the initiation of the Addiction Consult Service in 2019, Dauphin County Department of Drug and Alcohol has reported an increased use of their services within Penn State Health Medical Center (Table 8). In fact, they indicate that their response numbers since 2019 have increased every year. This has been attributed to increased awareness of substance use treatment modalities and resources by hospital staff through more frequent interactions with Addiction Consult Service members.

**Table 8:** Warm Handoff (WHO) Usage in Dauphin County Warm Handoff is a program through the Dauphin County Department of Drug and Alcohol, which aims to improve the health outcomes for patients who receive emergency medical care for opioid overdose by facilitating a seamless transition from the emergency department setting to specialty substance use disorder (SUD) treatment.

Date Range	Number of Patients Using Services (n)
July 2020-July 2021	69
July 2021-July 2022	73
July 2022-July 2023	91
July 2023-July 2024	132

When initiated after a nonfatal overdose, buprenorphine has been shown to increase abstinence and decrease future hospitalization rates, ED visits, health care dollars spent, and all-cause mortality [10]. Initiating treatment for opioid use disorder within the ED encounter has been shown to further positively impact rates of disease remission. A randomized clinical trial out of Yale School of Medicine showed that 78 percent of patients presenting for opioid dependence who initiated buprenorphine treatment within their emergency encounter were still engaged in SUD treatment 30 days later, compared to only 37 percent of the patients who were referred to outpatient resources only [11]. This data strongly supports the notion that in-hospital initiation of treatment for opioid use disorder is preferred to referral alone.

The evaluation of a similar service to the one offered at Penn State Health was completed at Vanderbilt University Medical Center in 2023. This specific service was found to increase MOUD initiation and reduce use of addiction services post-discharge for patients who received an addiction medicine consultation during their inpatient hospital treatment course. The study associated consultation service utilization with lower rates of return to the ED within 30 and 90 days [12]. Unlike the Vanderbilt study, which included only admitted patients, our analysis includes individuals evaluated in both inpatient and ED contexts. This broader scope allows for a more comprehensive understanding of the impact of addiction consultation services across the full spectrum of patient acuity and complexity.

Limitations

One major limitation was the single health system electronic medical record used to track patients following hospital discharge. We were able to quantify the patient return rate to Hershey Medical Center Emergency Department, but not whether they returned for substance intoxication at another hospital facility. Also, if patients continued to use following discharge but did not present to the ED, we would have falsely assumed these patients abstained from drug use.

Additionally, we did not have a way to confirm that patients who were referred to outpatient or inpatient services following discharge actually received such services. This would have required involving many of the local treatment facilities and being granted access to their medical records. For the study, we assumed that a patient who expressed consent to the treatment plan per the Addiction Consult Service note followed through with such a treatment plan. Patients who were sent to inpatient treatment facilities were closely monitored for the time they received treatment in the facility. With a limited follow-up window of 90 days, we may not have a full understanding of how the treatment plan impacted future drug use behavior. 

This study served as an objective evaluation of the current Addiction Consult Service program. The study was limited in its retrospective nature. Future studies should aim to obtain consent from patients in the hospital setting to evaluate the nature of their SUD prospectively over a 30- or 90-day period. Additionally, the medical research team should collaborate with local public health services to track patient outcomes after discharge.

## Conclusions

The benefit provided by early initiation of substance use disorder treatment in decreasing short-term ED return has been made clear in evaluating the consultation service available at Penn State Health. This allows for greater continuity of care, as well as the development of a more informed and personalized approach to treatment for the patient. Programs such as the Addiction Consult Service lead to improvement in healthcare resource allocation, which ultimately improves patient health outcomes and experiences.

These data confirm previous studies demonstrating the efficacy of ED treatment referral. As evidence continues to accumulate on the value of addiction consultation in improving outcomes for patients with substance use disorders, it becomes essential to expand and refine these services. This is especially critical in the ED setting, where many patients first present. Addiction consultation in the Emergency Department is an effective intervention strategy to initiate treatment for drug and alcohol use disorders and is associated with fewer ED visits in the subsequent 30 days.
